# Supplementary material for: Use of Antiplatelet Agents Decreases the Positive Predictive Value of Fecal Immunochemical Tests for Colorectal Cancer but Does Not Affect Their Sensitivity
Source: J Pers Med. 2021 Jun 1;11(6):497. doi: 10.3390/jpm11060497 (PMC8227279; doi:10.3390/jpm11060497)
Supplement: Supplementary file 1 [file jpm-11-00497-s001.zip › jpm-1179952-supplementary.pdf]

Appendix 1. Details of drug types in dual and triple therapies

Other dual therapy
Clopidogrel+Cilostazol N=3,866, Aspirin+Limaprost N=3,378, Aspirin+Triflusal N=2,945, Aspirin+Beraprost N=1,804, Aspirin+Ticlopidine N=1,302, Clopidogrel+Limaprost N=1,119, Triflusal+Clopidogrel N=1,100, Triflusal+Cilostazol N=839, Clopidogrel+Beraprost N=771, Indobufen+Clopidogrel N=651, Aspirin+Indobufen N=538, Cilostazol+Limaprost N=506, Cilostazol+Beraprost N=326, Triflusal+Limaprost N=285, Indobufen+Cilostazol N=227, Triflusal+Beraprost N=197, Ticlopidine+Cilostazol N=149, Beraprost+Limaprost N=143, Aspirin+Dipyridamole N=139, Indobufen+Limaprost N=125, Aspirin+Anagrelide N=101, Triflusal+Indobufen N=98, Indobufen+Beraprost N=90, Ticlopidine+Clopidogrel N=90, Triflusal+Ticlopidine N=80, Indobufen+Ticlopidine N=68, Ticlopidine+Limaprost N=40, Clopidogrel+Anagrelide N=28, Clopidogrel+Dipyridamole N=20, Ticlopidine+Beraprost N=19, Indobufen+Dipyridamole N=13, Cilostazol+Dipyridamole N=6, Triflusal+Dipyridamole N=4, Cilostazol+Anagrelide N=4, Dipyridamole+Beraprost N=4, Triflusal+Anagrelide N=2, Ticlopidine+Anagrelide N=1, Ticlopidine+Dipyridamole N=1, Dipyridamole+Limaprost N=1

Triple therapy
Aspirin+Clopidogrel+Cilostazol N=2,271, Aspirin+Clopidogrel+Limaprost N=291, Aspirin+Clopidogrel+Beraprost N=171, Aspirin+Triflusal+Clopidogrel N=170, Aspirin+Cilostazol+Limaprost N=148, Aspirin+Cilostazol+Beraprost N=121, Aspirin+Indobufen+Clopidogrel N=71, Aspirin+Triflusal+Cilostazol N=64, Clopidogrel+Cilostazol+Limaprost N=53, Aspirin+Ticlopidine+Cilostazol N=45, Triflusal+Clopidogrel+Cilostazol N=42, Clopidogrel+Cilostazol+Beraprost N=37, Aspirin+Beraprost+Limaprost N=33, Aspirin+Triflusal+Limaprost N=33, Clopidogrel+Beraprost+Limaprost N=21, Aspirin+Indobufen+Cilostazol N=20, Indobufen+Clopidogrel+Cilostazol N=17, Aspirin+Ticlopidine+Clopidogrel N=16, Aspirin+Triflusal+Beraprost N=15, Indobufen+Clopidogrel+Limaprost N=13, Aspirin+Clopidogrel+Anagrelide N=10, Triflusal+Clopidogrel+Limaprost N=10, Aspirin+Indobufen+Limaprost N=9, Aspirin+Ticlopidine+Beraprost N=9, Aspirin+Ticlopidine+Limaprost N=9, Triflusal+Clopidogrel+Beraprost N=9, Triflusal+Cilostazol+Limaprost N=9, Indobufen+Cilostazol+Limaprost N=9, Indobufen+Clopidogrel+Beraprost N=8, Cilostazol+Beraprost+Limaprost N=8, Aspirin+Triflusal+Ticlopidine N=6, Aspirin+Clopidogrel+Dipyridamole N=6, Aspirin+Cilostazol+Anagrelide N=6, Triflusal+Cilostazol+Beraprost N=6, Aspirin+Triflusal+Indobufen N=5, Aspirin+Indobufen+Ticlopidine N=5, Triflusal+Ticlopidine+Cilostazol N=5, Indobufen+Beraprost+Limaprost N=5, Indobufen+Cilostazol+Beraprost N=5, Aspirin+Indobufen+Beraprost N=4, Triflusal+Beraprost+Limaprost N=4, Indobufen+Ticlopidine+Clopidogrel N=3, Ticlopidine+Clopidogrel+Cilostazol N=3, Ticlopidine+Cilostazol+Beraprost N=3, Aspirin+Indobufen+Dipyridamole N=2, Triflusal+Indobufen+Clopidogrel N=2, Triflusal+Indobufen+Cilostazol N=2, Triflusal+Ticlopidine+Limaprost N=2, Triflusal+Ticlopidine+Clopidogrel N=2, Ticlopidine+Clopidogrel+Limaprost N=2, Clopidogrel+Cilostazol+Anagrelide N=2, Clopidogrel+Dipyridamole+Limaprost N=2, Cilostazol+Dipyridamole+Limaprost N=2, Aspirin+Triflusal+Anagrelide N=1, Aspirin+Triflusal+Dipyridamole N=1, Aspirin+Indobufen+Anagrelide N=1, Aspirin+Ticlopidine+Dipyridamole N=1, Aspirin+Cilostazol+Dipyridamole N=1, Aspirin+Dipyridamole+Beraprost N=1, Triflusal+Indobufen+Ticlopidine N=1, Triflusal+Cilostazol+Dipyridamole N=1, Indobufen+Ticlopidine+Beraprost N=1, Indobufen+Ticlopidine+Cilostazol N=1, Ticlopidine+Clopidogrel+Beraprost N=1, Ticlopidine+Cilostazol+Limaprost N=1, Clopidogrel+Anagrelide+Beraprost N=1

Table S1. Baseline characteristics in the entire study population.

	Non-users N = 4,638,167	Antiplatelet agents users						Anticoagulant users
		Antiplatelet agent, total N = 768,733	Antiplatelet monotherapy N = 701,683	Aspirin monotherapy N = 482,664	Clopidogrel monotherapy N= 98,468	Dual antiplatelet therapy N = 63,211	Triple antiplatelet therapy N = 3839	Warfarin N = 19,569
Sex								
Male	2,045,017 (44.1)	372,380 (48.4)	332,076 (47.3)	227,392 (47.1)	50,190 (51.0)	37,642 (59.6)	2662 (69.3)	10,715 (54.8)
Female	2,593,150 (55.9)	396,353 (51.6)	369,607 (52.7)	255,272 (52.9)	48,278 (49.0)	25,569 (40.5)	1177 (30.7)	8854 (45.3)
Age (years)	59.6 ± 7.9	64.9 ± 8.2	64.8 ± 8.2	64.4 ± 8.2	66.2 ± 8.3	66.4 ± 8.0	66.5 ± 7.9	65.4 ± 8.2
50–59	2,548,751 (55.0)	208,758 (27.2)	194,844 (27.8)	142,694 (29.6)	21,795 (22.1)	13,157 (20.8)	757 (19.7)	4862 (24.9)
60–69	1,456,611 (31.4)	315,607 (41.1)	288,162 (41.1)	198,720 (41.2)	39,629 (40.3)	25,838 (40.9)	1607 (41.9)	8002 (40.9)
70–79	564,055 (12.2)	213,178 (27.7)	190,786 (27.2)	123,856 (25.7)	31,726 (32.2)	21,102 (33.4)	1290 (33.6)	5881 (30.1)
≥ 80	68,750 (1.5)	31,190 (4.1)	27,891 (4.0)	17,394 (3.6)	5318 (5.4)	3114 (4.9)	185 (4.8)	824 (4.2)

Values are presented as the number (%) or mean ± SD. *P* values are compared between users and non-users with respect to each drug.
